# Ye Editor’s Vacation

**Published:** 1900-09

**Authors:** 


### Ye Editor's Vacation.

**DOWN ON THE FARM.**

"Give us a rest, oh, give us a rest," I sighed. Well, we've had a rest, and during the time have done some hard work. We havn't exactly experienced James Whitcomb Riley's "Knee-deep in June," but have lazed during the sweltering days of August, lolling on grassy lawns, beneath breezy trees-es, and have read all the late novels (that was the hard work). In the cool of the afternoon with the meek and lowly earth worm we'd angle for the frolicsome mud-cat, or beguile the festive perch in the turbid waters of the Colorado close by the ranch where we ranched; have watched the sun set, reveled in the delights of sixty-pound watermelons, and eaten of yaller-leg-pullets (fryin' size), great store. We plucked the luscious "Chinese-clings" and other sun-kissed peachy-cheeked clingers, to say nothing of cantaloupes. We have strolled down the meadow,—late of evenings—and watched the whistling mower as he mowed the Johnson-grass, and tumbled on the mounds of new-mown hay, like a school-boy, repeating, the meantime, those beautiful lines of Alfred Austin's: "Maud Muller's brother Jake, raked the meadow with a two-mule-rake." Oh, it's great. It makes a fellow poetic. Afternoons we would stroll down to the river bridge and see the picture Mrs. Townsend so beautifully describes of a summer day in the country:

"The soft wandering gale fills a silvery sail
That idly floats by on yon far-away stream;
A frail spirit-boat 'neath the other doth float,
Faintly fair, like some beautiful dream of a dream."

It was a big thing, too, to see

"The bee from the bosom of the red-clover blossom,
As he hurried to sip of the buck-wheat in bloom;
While the down of the thistle and the black-birds' clear whistle,
Were blent with the summer-day's light and perfume."

But the swelling on our editorial chin was bigger, where one of those same bumble-bees got in his work once, while I was trespassing on his preserves,—his favorite figs (fig preserves), which he and his were putting up expressly for family use. Much has been said and sung of the charms of pastoral life, and the milk-maid in her dainty cap has been an idyl,—an ideal which even queens have delighted to personate. Marie Antoinette didn't know a thing about milk-maids; she ought to have seen ours, out on King's Ranch. In this instance "she" was a big fat fellow with a sun-hurter straw hat and one gallus. To see him milk was not an idyl; nor was it an idle speculation, but more than a suspicion, that the milk was sterilized or fertilized by great drops of perspiration which gathered on the old fellow's brow about the size of a biscuit. Mrs. Townsend sang of

> "Brownie and Daisy, milk-laden and lazy,
> And the gentle-eyed heifer half standing aloof,
> While the dew-dimpled grass softly yields as they pass
> To the lingering print of each slowly-raised hoof."

That's real pretty,—isn't it? Our Brownie was a white cow,—a muley, and she was a kicker from away back. When she'd kick the milk-maid with the gallus and straw hat he'd kick her back. There was an alleged wag on the ranch, who was of a poetic turn. With his corn-cob pipe in his mouth, he'd saunter idly about, wishing something would happen; and when it did happen,—when the big ærolite passed over one night, he dodged and ran under the cow-shed. He'd say: "Doctor, come, come into the deep-tangled wild-wood, and let us see the cornucopiæs cope and the honey-suckles suck." Not much I would; too much exertion. But I stooped to bandy words with him, for which I despise myself. It were pleasanter, I told him, to loll on the broad verandas in a hammock, and listen to the lilacs lie; or stroll into the meadow, and in the gloaming listen to the cornstalk, and hear the May-pops pop; while the dear little grasshoppers sang, "In this Wheat Bye and Bye." Alas, that all things fair and bright must fade. Summer has gone, and already the bumble bees are humming of autumn days coming. Soon the frost will be upon the pumpkins and they will cease to pump. Back, now, to the harness and the mill, to grind out the despised but indispensable and ever elusive dollar, which no family can afford to be without.

